# Nationwide Implementation of Neoadjuvant Nivolumab-Based Chemo-Immunotherapy for Resectable NSCLC: Surgical Outcomes from a Prospective Multicenter Study

**DOI:** 10.1245/s10434-026-20310-2

**Published:** 2026-07-30

**Authors:** Jiri Vachtenheim Jr, Fernando Casas-Mendez, Rene Novysedlak, Janis Tavandzis, Linda Capkova, Jaromir Vajter, Marek Szkorupa, Juraj Kultan, Alice Taskova, Martina Koziar Vasakova, Martin Skala, Martin Svaton, Vilem Maly, David Sulc, Ivo Hanke, Michal Hrnciarik, Ladislav Mitas, Monika Bratova, Lubomir Tulinsky, Radoslava Cernekova, Zuzana Ozaniak Strizova, Matyas Kuhn, Boris Sepesi, Jitka Palich Fucikova, Robert Lischke

**Affiliations:** 1 3rd Department of Surgery, First Faculty of Medicine, Charles University and University Hospital Motol and Homolka, Prague, Czech Republic; 2 Pneumology and Oncology Departments, Second Faculty of Medicine, Charles University and Center for Thoracic Oncology and Surgery, University Hospital Motol and Homolka, Prague, Czech Republic; 3 Department of Pathology and Molecular Medicine, Second Faculty of Medicine, Charles University and University Hospital Motol and Homolka, Prague, Czech Republic; 4 Department of Anesthesiology and Intensive Care Medicine, Second Faculty of Medicine, Charles University and University Hospital Motol and Homolka, Prague, Czech Republic; 5 University Hospital Olomouc and Faculty of Medicine and Dentistry, Palacký University Olomouc, Olomouc, Czech Republic; 6 Department of Pulmonary Diseases and Tuberculosis, University Hospital Olomouc and The Faculty of Medicine of Palacký University in Olomouc, Olomouc, Czech Republic; 7 Department of Thoracic Surgery 3rd Faculty of Medicine, Charles University and Thomayer University Hospital, Prague, Czech Republic; 8 Department of Pneumology, First Faculty of Medicine, Charles University and Thomayer University Hospital, Prague, Czech Republic; 9 Department of Surgery, Charles University and University Hospital, Pilsen, Czech Republic; 10 Department of Pneumology and Phthiseology, Faculty of Medicine in Pilsen, University Hospital in Pilsen, Charles University, Pilsen, Czech Republic; 11 Department of Thoracic Surgery, Krajska zdravotni a.s. and UJEP, Ústí nad Labem, Czech Republic; 12 Department of Clinical Oncology, Krajská Zdravotní Masaryk Hospital, Ústí nad Labem, Czech Republic; 13 Department of Cardiac Surgery, Faculty of Medicine, Charles University in Hradec Králové and University Hospital Hradec Králové, Hradec Králové, Czech Republic; 14 Department of Pneumology, Faculty of Medicine in Hradec Kralove, University Hospital Hradec Kralove, Czech Republic, Charles University, Prague, Czech Republic; 15 Department of Surgery, University Hospital Brno and Faculty of Medicine, Masaryk University, Brno, Czech Republic; 16 Department of Respiratory Diseases, University Hospital Brno, Department of Comprehensive Cancer Care, Masaryk Memorial Cancer Institute, Faculty of Medicine, Masaryk University, Brno, Czech Republic; 17 Department of Surgery, University Hospital Ostrava, Ostrava, Czech Republic; 18 Department of Oncology, University Hospital Ostrava, Ostrava, Czech Republic; 19 Department of Immunology, Second Faculty of Medicine, Charles University and University Hospital Motol and Homolka, Prague, Czech Republic; 20 ANOVA CRO s.r.o., Brno, Czech Republic; 21 Sarah Cannon Cancer Network, Swedish Medical Center, Englewood, CO USA; 22 Sotio Biotech, Prague, Czech Republic

**Keywords:** Non-small cell lung cancer, Neoadjuvant chemo-immunotherapy, Nivolumab, Thoracic surgery, Surgical outcomes, Pathological response

## Abstract

**Background:**

Neoadjuvant chemo-immunotherapy has transformed the treatment landscape for resectable non-small-cell lung cancer (NSCLC). However, real-world surgical data from nationwide European cohorts remain limited. This study evaluated surgical outcomes and practice patterns during the first year of nationwide implementation of neoadjuvant chemo-immunotherapy with nivolumab in the Czech Republic.

**Methods:**

This prospective, multicenter, observational cohort study included all consecutive patients with resectable programmed cell death ligand-1(PD-L1) ≥ 1% NSCLC treated according to the CheckMate 816 protocol (platinum-based chemotherapy plus nivolumab) at all eight accredited thoracic oncology centers in the Czech Republic between August 2023 and August 2024. Surgical and pathological outcomes were assessed.

**Results:**

Sixty-four patients initiated neoadjuvant therapy and 56 (87.5%) underwent resection. Minimally invasive surgery was attempted in 46.4% of cases, with an 11.5% conversion rate. Clavien–Dindo grade ≥ III complications occurred in 14.3%, with grade IIIb representing the highest severity. 30-day mortality was 0%. Although 90-day mortality was 5.4%, none of the deaths were directly attributable to surgery. R0 resection was achieved in 98.2%. In the intention-to-treat population, pathological complete response and major pathological response rates were 40.6% and 48.4%, respectively. Pathological complete response was significantly higher in squamous than in non-squamous NSCLC (66.7% vs. 27.6%; *p *= 0.003).

**Conclusions:**

In this first nationwide cohort from a European country, early adoption of neoadjuvant chemo-immunotherapy with nivolumab in patients with resectable PD-L1 ≥ 1% NSCLC was associated with acceptable perioperative outcomes, high R0 resection rates, and substantial pathological response. These results demonstrate the successful adoption of a novel NSCLC treatment modality across a nationwide healthcare system.

**Supplementary Information:**

The online version contains supplementary material available at https://doi.org/10.1245/s10434-026-20310-2.

Lung cancer remains the leading cause of cancer-related mortality worldwide, and non-small-cell lung cancer (NSCLC) accounts for approximately 85% of all lung cancer cases.^1^ In medically fit patients, surgical resection with systematic lymph node dissection is the cornerstone of treatment for early-stage NSCLC.^2^ However, patients with resectable disease at the time of diagnosis represent merely 20–30%.^3^ Historically, patients with stage III disease (8th edition of the American Joint Committee on Cancer [AJCC]) who underwent radical surgical resection with curative intent experienced 5-year recurrence-free survival of only 37%. Five-year overall survival has remained poor for all subgroups of stage III disease, ranging from 36 to 26% and 13% in stages IIIA, IIIB, and IIIC, respectively.^4,5^ The addition of chemotherapy to surgery in the neoadjuvant or adjuvant setting has provided only a modest overall survival benefit of approximately 5%.^6,7^

Recently, several phase III randomized clinical trials have demonstrated substantially improved oncological outcomes in patients with resectable NSCLC treated with neoadjuvant or perioperative chemo-immunotherapy compared with neoadjuvant chemotherapy alone. These improvements were observed across multiple endpoints, including event-free survival (CheckMate816, KEYNOTE-671, AEGEAN, and CheckMate77T), overall survival (CheckMate816 and KEYNOTE-671), and pathological response measures such as pathological complete response (pCR) and major pathological response (MPR).^8–12^ Results from these trials have led to an unprecedented transformation of the treatment paradigm for resectable, including locally advanced, NSCLC.^13^ The CheckMate816 study protocol (three cycles of neoadjuvant platinum-based chemotherapy plus nivolumab) became the first neoadjuvant chemo-immunotherapy regimen approved by the European Medicines Agency (EMA) in 2023.^14^ However, the implementation of this treatment regimen varied across European countries according to national regulatory approval processes.^15^

In 2025, overall survival data from CheckMate816 were published, demonstrating a 5-year overall survival of 65.4% in the chemo-immunotherapy arm and 95.3% in the subgroup of patients who achieved pCR.^12^ Randomized clinical trials in lung cancer represent a highly controlled environment with rigorous patient selection, which may not fully reflect all the complexities of routine clinical practice, including the treatment of older and more comorbid patients.^16^ Accordingly, the generalizability of results from randomized clinical trials to unselected real-world populations remains uncertain. Additionally, CheckMate816 and other pivotal trials did not focus on reporting detailed data on perioperative patient management, surgical techniques or conduct of operations based on intraoperative findings. These variables may also influence perioperative outcomes and daily thoracic surgical practice.^13^ There remains an unmet need for reporting real-world surgical outcomes outside of clinical trials, particularly during the initial adoption phase of novel treatment strategies into routine practice.^17^

Only a limited number of studies have reported real-world data on surgical outcomes in patients treated with neoadjuvant chemo-immunotherapy with nivolumab since the EMA approval in March 2023. In Europe, one retrospective surgical series from England (*n *= 130) and a recent multicenter retrospective study (*n *= 340) have reported surgical outcomes after neoadjuvant chemo-immunotherapy with nivolumab.^18,19^

Nivolumab-based neoadjuvant chemo-immunotherapy became available in the Czech Republic through an early-access program in August 2023, although routine reimbursement was not introduced until July 2024. This transition period enabled the evaluation of an early nationwide implementation of this treatment strategy into routine clinical practice. The aim of this study was to provide nationwide real-world data on surgical outcomes in patients treated with neoadjuvant chemo-immunotherapy with nivolumab during the first year of clinical implementation in the Czech Republic.

## Methods

### Study Design and Overview

This prospective, observational, multicenter cohort study was designed to provide comprehensive nationwide data on surgical outcomes and clinical practice patterns in patients with resectable NSCLC following neoadjuvant chemo-immunotherapy (nivolumab) during the first year of its availability in the Czech Republic (August 2023–August 2024), with follow-up censored on January 31, 2025. The study was conducted in collaboration with all eight thoracic surgical oncology centers designated by the Czech Ministry of Health at the time of study initiation.

Nivolumab was initially provided through a manufacturer-funded early access program prior to standard reimbursement, with the timing of treatment initiation varying across participating institutions because of administrative factors. The study was approved by the institutional ethics committee at the principal investigator’s institution (University Hospital Motol; reference number EK-410/23). Prior to patient enrolment, all participating centers obtained approval from their local institutional ethics committees, including permission to share deidentified data with the coordinating center. All patients provided written informed consent prior to treatment initiation. Clinical decisions regarding treatment, diagnostics, and follow-up remained entirely at the discretion of treating physicians.

### Study Population and Clinical Management

All patients with European Cooperative Oncology Group (ECOG) performance status 0–1 and resectable NSCLC treated with neoadjuvant chemo-immunotherapy according to the CheckMate816 protocol during the study period were included.^8^ In the Czech Republic, eligibility for this treatment was restricted, according to the EMA approval, to patients with clinical stage IIB–IIIB disease (N2 only) based on the 8th edition of the AJCC staging system, tumor programmed death ligand-1 (PD-L1) expression ≥ 1%, and the absence of epidermal growth factor receptor mutations or anaplastic lymphoma kinase alterations.

The detailed treatment pathway is reported in the Supplementary data. In general, all patients were discussed at multidisciplinary tumor boards, and resectability was confirmed by board-certified thoracic surgeons prior to treatment initiation. Surgery was planned within 4–6 weeks after the third dose of neoadjuvant chemo-immunotherapy. Perioperative variables, including technical complexity, were collected. Pathological outcomes included pCR, defined as 0% viable cells in the resected specimen, including the primary tumor and lymph nodes, and MPR, defined as 0–10% residual viable tumor.

### Statistical Analysis

The intention-to-treat (ITT) population comprised all patients who initiated neoadjuvant chemo-immunotherapy, irrespective of subsequent surgical resection. The variables that were collected and used for the analysis are reported in the Supplementary data. Race and ethnicity data were not collected because these variables are not routinely recorded as part of standard clinical documentation across participating centers in the Czech Republic. Tables provide a descriptive summary of the study population. Pre-surgical data were stratified by whether patients underwent surgery or not. Continuous variables were summarized using the mean, median, interquartile range (IQR), and first and third quartiles. Categorical variables were summarized as counts and percentages based on available data. Selected patient subgroups were compared according to the pCR and MPR after surgery. Differences between subgroups were assessed using Pearson’s chi-squared test. The relationship between preoperative clinical staging and postoperative pathological staging was visualized using Sankey diagrams. All statistical analyses were performed using SAS software, version 9.4 (SAS Institute, Cary, NC, USA). A two-sided *p*-value < 0.05 was considered statistically significant.

## Results

### Patient Characteristics

A total of 64 patients initiated neoadjuvant platinum-based chemotherapy combined with nivolumab for resectable NSCLC across eight academic thoracic surgical oncology centers in the Czech Republic between August 2023 and August 2024. Baseline patient characteristics are summarized in Table 1. The median age at diagnosis was 69.5 years (IQR 63.0–74.0), and 43 patients (67.2%) were older than 65 years. Thirty-six patients (56.2%) were males. Squamous cell carcinoma was the most frequent histological subtype, observed in 31 patients (48.4%). A total of 44 patients (68.8%) had clinical stage III disease. High PD-L1 expression (≥ 50%) was detected in 29 cases (45.3%).

**Table 1:** Baseline patient characteristics

Characteristic	Pts with surgery (*n *= 56)	Pts without surgery (*n *= 8)	Overall (*n *= 64)
Sex			
Male	30 (53.6)	6 (75)	36 (56.2)
Female	26 (46.4)	2 (25)	28 (43.8)
ECOG-PS			
0	30 (53.6)		30 (46.9)
1	26 (46.4)	8 (100)	34 (53.1)
Smoking status			
Current	29 (51.8)	3 (37.5)	32 (50)
Former	22 (39.3)	5 (62.5)	27 (42.2)
Never	5 (8.9)		5 (7.8)
History of malignancy	14 (25)	1 (12.5)	15 (23.4)
History of previous thoracic surgery	3 (5.4)	1 (12.5)	4 (6.3)
BMI, kg/m^2^	27.4 (24.7–30.7)	27.30 (25.7–30.0)	27.20 (24.8–30.0)
Age at biopsy, years	68.5 (61.5–74)	74 (69–75.5)	69.5 (63–74)
Histological type			
Squamous-cell carcinoma	25 (44.6)	6 (75)	31 (48.4)
Adenocarcinoma	27 (48.2)	2 (25)	29 (45.4)
Adenosquamous carcinoma	2 (3.6)		2 (3.1)
Not otherwise specified	2 (3.6)		2 (3.1)
Tumor size, mm	54 (45–61.5)	63.5 (54–76.5)	55 (48–64.5)
Clinical TNM classification			
cT			
T1b	1 (1.8)		1 (1.6)
T1c	5 (8.9)		5 (7.8)
T2a	4 (7.1)		4 (6.3)
T2b	7 (12.5)		7 (10.9)
T3	26 (46.4)	6 (75)	32 (50)
T4	13 (23.2)	2 (25)	15 (23.4)
cN			
N0	20 (35.7)	2 (25)	22 (34.4)
N1	15 (26.8)	3 (37.5)	18 (28.1)
N2	21 (37.5)	3 (37.5)	24 (37.5)
cM: M0	56 (100)	8 (100)	64 (100)
Disease stage			
IIB	18 (32.1)	2 (25)	20 (31.2)
IIIA	28 (50)	4 (50)	32 (50)
IIIB	10 (17.9)	2 (25)	12 (18.8)
PD-L1 TPS, %	35 (5–80)	67.5 (15–87.5)	37.5 (5–80)
PD-L1 category			
1–49 %	32 (57.1)	3 (37.5)	35 (54.7)
≥ 50 %	24 (42.9)	5 (62.5)	29 (45.3)

Categorical variables are expressed as n (%); continuous variables are presented as median (interquartile range). Available data are reported for each variable, and percentages are calculated from the number of patients without missing values within the respective group. Age refers to age at the time of biopsy. Clinical TNM classification is based on pretreatment assessment. PD-L1 TPS is presented as a continuous variable and categorically (1–49% and ≥ 50%)

BMI, body mass index; ECOG-PS, Eastern Cooperative Oncology Group performance status; n, number of patients without missing values; pts, patients; PD-L1, programmed cell death ligand-1; TNM, tumor–node–metastasis; TPS, tumor proportion score

### Neoadjuvant Treatment and Radiologic Response

In total, 63 patients (98.4%) completed all three planned cycles of neoadjuvant chemo-immunotherapy. One patient (1.6%) received only two cycles because of tumor abscess complicated by pneumonia and concomitant herpetic infection. Neoadjuvant treatment-related adverse events precluding surgery occurred in two patients (3.1%). One patient presented with immune-related pneumonitis, and another developed severe cardiac dysfunction attributed to chemotherapy.

Restaging imaging was performed in 63 patients (98.4%). One patient (1.6%) did not undergo restaging because they died from severe COVID-19—associated pneumonia during the third treatment cycle. Among patients undergoing restaging, positron emission tomography/computed tomography (PET/CT) was the most frequently used modality (63.5%), followed by contrast-enhanced CT (31.7%). In three patients (4.8%), chest radiography was performed for logistical reasons; these patients were not evaluable for radiologic response. Among 60 evaluable patients, partial response was observed in 49 cases (81.7%); three patients had disease progression. Despite radiologic progression, two of the three patients were considered technically resectable and underwent surgery (Tables 2 and 3).

**Table 2:** Neoadjuvant treatment and radiological response

Characteristic	Pts with surgery (*n *= 56)	Pts without surgery (*n *= 8)	Overall (*n *= 64)
Type of chemotherapy			
CBDCA+PTX	46 (82.1)	7 (87.5)	53 (82.8)
cDDP+PEM	8 (14.3)	1 (12.5)	9 (14.1)
CBDCA+PEM	2 (3.6)		2 (3.1)
Total number of cycles			
2	1 (1.8)		1 (1.6)
3	55 (98.2)	8 (100)	63 (98.4)
*TRAE leading to surgery delay*	*3 (5.4)*		
*Nephrotoxicity*	*1 (33.3)*		
*Dehydration*	*1 (33.3)*		
*Immune-related pneumonitis*	*1 (33.3)*		
Type of restaging imaging			
n/missing	56/0	7/1^a^	63/1^a^
CT	17 (30.4)	3 (42.9)	20 (31.7)
PET/CT	36 (64.2)	4 (57.1)	40 (63.5)
CXR	3 (5.4)		3 (4.8)
Tumor response status			
n/missing	56/0	7/1^a^	63/1^a^
Non-progression	1 (1.8)		1 (1.6)
Progression	2 (3.6)	1 (14.3)	3 (4.8)
Regression	53 (94.6)	6 (85.7)	59 (93.7)
Nodal response status			
n/missing	52/4	6/2^a^	58/6^a^
Non-progression	15 (28.8)	1 (16.7)	16 (27.6)
Progression	3 (5.8)		3 (5.2)
Regression	34 (65.4)	5 (83.3)	39 (67.2)
RECIST/PERCIST according to CT or PET/CT			
n/missing	53/3	7/1^a^	60/4^a,b^
CR	6 (11.3)		6 (10)
PR	43 (81.1)	6 (85.7)	49 (81.7)
SD	2 (3.8)		2 (3.3)
PD	2 (3.8)	1 (14.3)	3 (5)

Categorical variables are expressed as n (%); available data are reported for each variable, and percentages are calculated from the number of patients without missing values within the respective group. Detailed descriptions of TRAE are provided only for affected patients. Tumor and nodal response statuses are based on restaging assessment. Radiologic response according to RECIST or PERCIST criteria was evaluated using CT or PET/CT, as applicable. For italicized subcategories, percentages are calculated using the total number of patients within the respective parent subgroup as the denominator

^a^One patient in the non-surgical group died from COVID-19—associated pneumonia 8 days after the administration of the third dose of neoadjuvant treatment and was therefore not restaged

^b^Three additional patients had missing restaging data because CT or PET/CT was not performed

CBDCA, carboplatin; cDDP, cisplatin; CR, complete response; CT, computed tomography; CXR, chest X-ray; n, number of patients without missing values.; PD, progressive disease; PEM, pemetrexed; PERCIST, Positron Emission Tomography Response Criteria in Solid Tumors; PET, positron emission tomography; PR, partial response; pts, patients; PTX, paclitaxel; RECIST, Response Evaluation Criteria in Solid Tumors; RTG, chest radiography; SD, stable disease; TRAE, treatment-related adverse event

**Table 3:** Failure to proceed to surgery, causes of cancellation or delay, and perioperative surgical data

Characteristics	Pts with surgery (*n *= 56)	Pts without surgery (*n *= 8)	Overall (*n *= 64)
Surgery			
Yes			56 (87.5)
No			8 (12.5)
Cause of surgery cancellation			
Decline in lung function and/or overall condition (not directly attributable to neoadjuvant treatment)		2 (25)	
Neoadjuvant treatment-related adverse events		2 (25)	
Death from severe COVID-19–associated pneumonia		1 (12.5)	
Patient refusal, lost to follow-up		1 (12.5)	
Progressive disease with superior vena cava infiltration		1 (12.5)	
Severe heart disease		1 (12.5)	
*Delayed surgery*	*22 (39.3)*		
*Cause of surgery delay*			
*Administrative reasons*	*9 (40.9)*		
*Infection*	*5 (22.7)*		
*Invasive staging for new finding on restaging*	*2 (9.1)*		
*Patient preference*	*2 (9.1)*		
*TRAEs—chemotherapy*	*2 (9.1)*		
*MDT discussion*	*1 (4.5)*		
*TRAEs—immunotherapy*	*1 (4.5)*		
*Surgery delay under 14 days*			
*n/delayed surgeries (%)*	*14/22 (63.6)*		
*Surgery delay over 14 days*			
*n/delayed surgeries (%)*	*8/22 (36.4)*		
Days between last dose and surgery	38 (31–50)		
Days between end of last cycle and surgery	17 (10–29)		
Intra-operatively resectable	56 (100)		
Duration of surgery, mins	167.5 (132–195)		
Type of surgical approach			
Open	30 (53.6)		
*RATS*	*18 (32.1)*		
*VATS*	*8 (14.3)*		
*Conversion from VATS to open surgery*			
*n/VATS (%)*	*2/8 (25)*		
*Conversion from RATS to open surgery*			
*n/RATS (%)*	*1/18 (5.6)*		
Extent of surgery performed			
Lobectomy	45 (80.4)		
Bilobectomy	5 (8.9)		
Pneumonectomy	5 (8.9)		
Wedge resection	1 (1.8)		
Side			
Right	38 (67.9)		
Left	18 (32.1)		
*Extended lung parenchyma resection*	*4 (7.1)*		
*Extent of additional resection*			
*Segmentectomy S6*	*2 (50)*		
*Wedge resection of middle and lower lobe*	*1 (25)*		
*Left atrium*	*1 (25)*		

Data are presented separately for patients who underwent surgery, patients who did not undergo surgery, and for the overall cohort, where applicable. Categorical variables are expressed as n (%); continuous variables are reported as median (interquartile range). Available data are reported for each variable, and percentages are calculated from the number of patients without missing values within the respective group. Failure to proceed to surgery includes patients who did not proceed to resection after completion of neoadjuvant therapy; causes of cancellation are specified for affected patients only. Causes of surgical delay are reported among patients with documented postponement. For italicized subcategories, percentages are calculated using the total number of patients within the respective parent subgroup as the denominator

MDT, multidisciplinary team; n, number of patients without missing values; pts, patients; RATS, robotic-assisted thoracic surgery; TRAEs, treatment-related adverse events; VATS, video-assisted thoracoscopic surgery

### Surgical Planning, Treatment, and Perioperative Outcomes

Following neoadjuvant treatment, eight (12.5%) patients did not undergo planned surgical resection. Except for the two patients (death from severe COVID-19–associated pneumonia and one patient’s withdrawal from the treatment), all individuals who did not undergo surgery received radiotherapy. The patient who died from COVID-19–associated pneumonia remained part of the ITT population but was not included in postoperative mortality analyses. Overall, 62 of 64 patients (96.9%) received locoregional therapy (either surgery or radiotherapy) after neoadjuvant chemo-immunotherapy. The median interval between the last dose of neoadjuvant chemo-immunotherapy and surgery was 38 days (IQR 31.0–50.0). Twenty-two patients (39.3%) underwent surgery more than 6 weeks after the last dose of neoadjuvant chemo-immunotherapy, and in the majority (63.6%) of cases the delay was < 2 weeks. The most frequent cause of the delay was administrative reasons (40.9%) (Table 3).

All patients who underwent surgery were deemed resectable during intraoperative exploration. More than half of the procedures were performed through thoracotomy (53.6%). A minimally invasive approach was used in 26 cases (46.4%), including robotic-assisted thoracic surgery (RATS) in 18 patients (32.1%) and video-assisted thoracoscopic surgery (VATS) in 8 patients (14.3%). Conversion to thoracotomy occurred in 3 of 26 minimally invasive operations (11.5%), including 1 of the 18 RATS (5.6%) and 2 of the 8 VATS procedures (25.0%) and was attributed to hilar fibrosis in all cases. The most frequently performed procedure was lobectomy (80.4%), and pneumonectomy was performed in five cases (8.9%). Bronchoplasty, angioplasty, or combined reconstruction was required in six patients (10.7%), chest wall resection in three patients (5.4%), and intrapericardial dissection in four patients (7.1%). Significant fibrosis and lymphadenopathy contributing to technical operative difficulties were reported in 25 (44.6%) of cases (Table 3 and Supplementary Table S1).

The median length of hospital stay was 7 days (IQR 5.0–10.0). At least one Clavien–Dindo grade ≥ III complication occurred in 8 patients (14.3%), including five grade IIIa (8.9%) and four grade IIIb events (7.1%). No postoperative complications were directly attributable to neoadjuvant chemo-immunotherapy. Pulmonary complications represented the majority of clinically significant postoperative adverse events, whereas cardiovascular complications were uncommon. The 30-day postoperative mortality was 0%. Within 30 days after discharge, a Clavien–Dindo grade ≥ III (IIIa) complication was observed in one patient (1.8%). The 90-day postoperative mortality was 5.4% (*n *= 3), with two deaths due to pneumonia and one due to heart failure (Table 4).

**Table 4:** Postoperative outcomes and surgery-related adverse events

Characteristic	Pts with surgery (*n *= 56)
Days with chest tube	4 (3–7)
Days in ICU	2 (1–3.5)
Discharged after surgery	56 (100)
Total hospital days	7 (5–10)
Patients with surgery-related AE	28 (50)
Captured surgery-related AEs during hospital stay	
Anemia	8 (14.3)
Prolonged air leak	7 (12.5)
Pleural effusion or pneumothorax requiring re-drainage	5 (8.9)
Subcutaneous emphysema	5 (8.9)
Pain	4 (7.1)
Pneumonia	4 (7.1)
Bronchopleural fistula	2 (3.6)
Chylothorax	2 (3.6)
Atelectasis requiring bronchoscopy	1 (1.8)
Atrial fibrillation	1 (1.8)
Immune-related pneumonitis	1 (1.8)
Middle lobe infarction	1 (1.8)
Other^a^	3 (5.4)
Clavien–Dindo AE classification during hospital stay	
Grade III event	8 (14.3)
Grade IIIa event	5 (8.9)
Grade IIIb event	4 (7.1)
Readmission during first 30 days after surgery (pleural effusion)	1 (1.8)

Data are presented for patients who underwent surgery. Continuous variables are expressed as median (interquartile range), and categorical variables as n (%). Available data are reported for each variable, and percentages are calculated from the number of patients without missing values. Postoperative length-of-stay parameters include duration of chest tube drainage, days in the intensive care unit, and total hospital stay. Surgery-related adverse events include any in-hospital complication considered attributable to the surgical procedure. Individual adverse events are reported by type. Severity was classified according to the Clavien–Dindo system; grade III denotes complications requiring surgical, endoscopic, or radiologic intervention (IIIa without general anesthesia; IIIb under general anesthesia). Thirty-day readmission refers to unplanned rehospitalization within 30 days after surgery; the reason for readmission is specified for affected patients only

^a^The category “Other” includes *Candida albicans* infection (sputum), electrolyte infusion, and mental deterioration resulting in drain disconnection

AE, adverse event; ICU, intensive care unit; n, number of patients without missing values; pts, patients

### Pathological Outcomes and Follow-Up

**Fig. 1:**
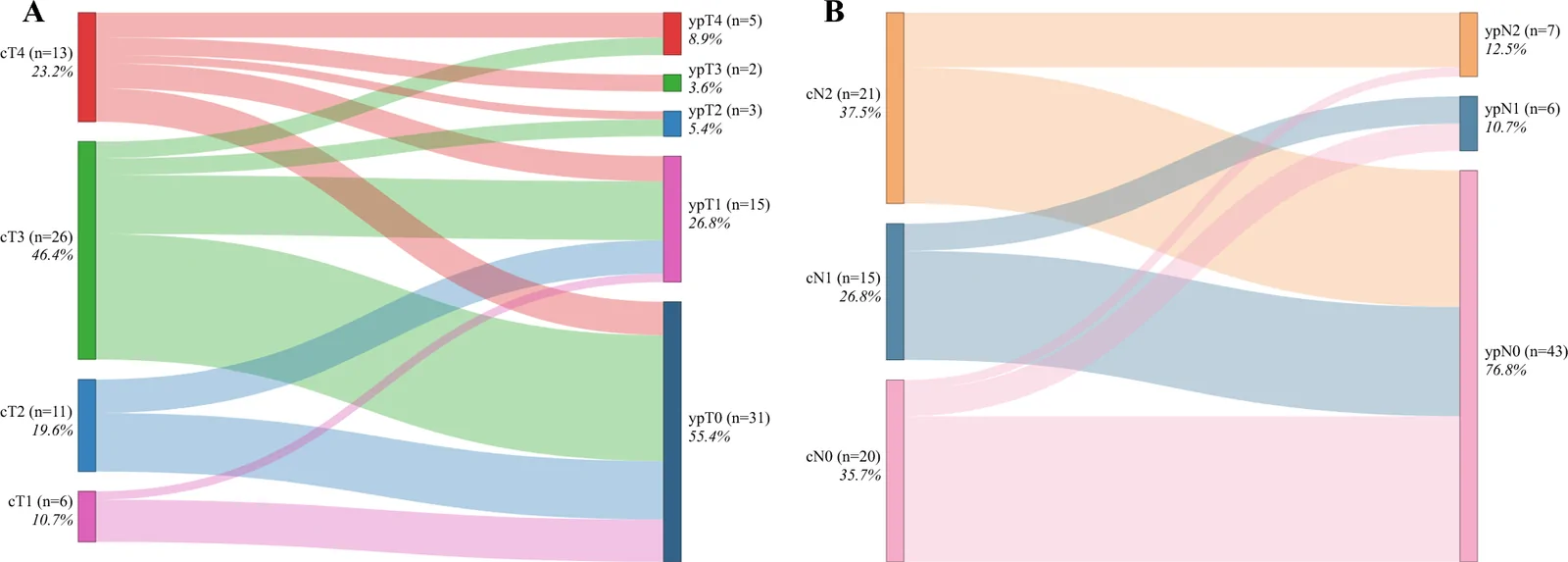
Tumor and nodal stage evolution: clinical staging before surgery and pathological staging after neoadjuvant chemo-immunotherapy and surgical resection. In both panels, the width of each flow is proportional to the number of patients transitioning from a given clinical stage to the corresponding pathological stage. Absolute numbers and percentages are shown relative to the total cohort. **A** Sankey diagram illustrating the relationship between clinical tumor stage (cT) assessed before surgery and post-treatment pathological tumor stage (ypT) determined after neoadjuvant therapy and surgical resection. Pathological stage ypT0 represents complete pathological response in the primary tumor. **B** Sankey diagram illustrating the relationship between clinical nodal stage (cN) assessed before surgery and post-treatment pathological nodal stage (ypN) determined after neoadjuvant therapy and surgical resection. Pathological nodal stage ypN0 represents the absence of residual nodal metastasis at definitive histopathological examination

R0 resection was achieved in 55 patients (98.2%). pCR was observed in 26 patients (46.4% of operated patients and 40.6% of the ITT population) and MPR in 31 patients (60.8% and 48.4%, respectively), with missing data in 5 patients, as the percentage of residual viable tumor was unavailable (Table 5). pCR and MPR rates did not differ significantly according to clinical stage (II vs. III; *p *= 0.71 and *p *= 0.87), PD-L1 expression (< 50% vs. ≥ 50%; *p *= 0.64 and *p *= 0.56), or clinical nodal status (*p *= 0.73 and *p *= 0.51). A significantly higher pCR rate was observed in squamous versus non-squamous histology (66.7% vs. 27.6%; *p *= 0.003), whereas the difference in MPR did not reach statistical significance (73.1% vs. 48.0%; *p *= 0.067) (Table 6). Pathological tumor size reduction following neoadjuvant chemo-immunotherapy was observed in 50 resected specimens (89.3%) compared with pre-treatment radiographical tumor measurement. Among 36 patients who underwent surgery with initially positive nodal status (cN1-2), pathological nodal downstaging to ypN0 was observed in 27 patients (75.0%). The evolution of tumor size and nodal involvement is shown in Fig. 1.

**Table 5:** Pathological endpoints

Characteristics	Pts with surgery (*n *= 56)
ypT	
T0	31 (55.4)
T1a	5 (8.9)
T1b	3 (5.4)
T1c	6 (10.6)
T1mi	1 (1.8)
T2a	2 (3.6)
T2b	1 (1.8)
T3	2 (3.6)
T4	5 (8.9)
ypN	
N0	43 (76.8)
N1	6 (10.7)
N2	7 (12.5)
Total number of examined LNs	18.5 (12.5–25)
Number of LN groups with metastases	
0	43 (76.8)
1	5 (8.9)
2	5 (8.9)
3	1 (1.8)
5	1 (1.8)
7	1 (1.8)
Completeness of resection	
R0	55 (98.2)
R1	1 (1.8)
pCR (ypT0 ypN)	26 (46.4)
MPR (ypT+ ypN0)	31 (60.8)^a^
ypT0 ypN1/ypN2	5 (8.9)

Pathological endpoints are presented for patients who underwent surgical resection following neoadjuvant therapy. ypT, ypN, completeness of resection, and response categories are expressed as n (%). Continuous variables, including number of examined LNs, number of metastatic LNs, and residual viable tumor, are reported as median (IQR). pCR is defined as ypT0 ypN0 (0% residual viable tumor in the resected specimen). MPR is defined according to established criteria (≤ 10% residual viable tumor in the resected specimen). Available data are reported for each variable, and percentages are calculated from the number of patients without missing values

^a^MPR analysis excludes patients from one center (Prague: Thomayer University Hospital, *n *= 5) because only binary results (tumor vs. no tumor) were available and percentage data on residual viable tumor could not be derived

IQR, interquartile range; LN, lymph node; MPR, major pathological response; n, number of patients without missing values; pCR, pathological complete response; pts, patients; R0/R1, resection margin status; ypN, post-treatment pathological nodal stage; ypT, post-treatment pathological tumor stage

**Table 6:** Pathological endpoints by subgroups

Groups	pCR			MPR^a^		
	n	Yes	No	n/missing	Yes	No
Disease stage						
II	18	9 (50)	9 (50)	16/2	10 (62.5)	6 (37.5)
III	38	17 (44.7)	21 (55.3)	35/3	21 (60)	14 (40)
*p*-value		0.712			0.865	
PD-L1 expression level (TPS)						
1–49 %	32	14 (43.8)	18 (56.3)	28/4	16 (57.1)	12 (42.9)
≥ 50 %	24	12 (50)	12 (50)	23/1	15 (65.2)	8 (34.8)
*p*-value		0.643			0.557	
Tumor histology						
Squamous including adenosquamous	27	18 (66.7)	9 (33.3)	26/1	19 (73.1)	7 (26.9)
Non-squamous	29	8 (27.6)	21 (72.4)	25/4	12 (48)	13 (52)
*p*-value		0.003			0.067	
Clinical nodal stage						
N0	20	8 (40)	12 (60)	18/2	10 (55.6)	8 (44.4)
N1	15	7 (46.7)	8 (53.3)	12/3	9 (75)	3 (25)
N2	21	11 (52.4)	10 (47.6)	21/0	12 (57.1)	9 (42.9)
*p*-value		0.729			0.512	

Pathological endpoints are presented for predefined clinical and tumor-related subgroups. pCR and MPR are expressed as n (%) within each subgroup. Available data are reported for each comparison, and percentages are calculated from patients without missing values in the respective subgroup. P-values refer to comparisons between categories within each subgroup. Statistical comparisons were performed using Pearson’s chi-squared test

^a^MPR analysis excludes patients from one center (Prague: Thomayer University Hospital, *n *= 5) because only binary results (tumor vs. no tumor) were available and percentage data on residual viable tumor could not be derived

MPR, major pathological response; n, number of patients without missing values; pCR, pathological complete response; PD-L1, programmed cell death ligand-1; TPS, tumor proportion score

With a median follow-up of 255.5 days (IQR 207.0–322.0) from treatment initiation, eight events were observed among 56 operated patients (14.3%), including three non–malignancy-related deaths within 90 days after surgery and five disease-related events, comprising one locoregional and four distant recurrences. All recurrences occurred in patients with incomplete pathological responses to neoadjuvant therapy. However, the limited follow-up period and low event count preclude drawing conclusions about recurrence patterns or survival outcomes in this study cohort.

## Discussion

Neoadjuvant chemo-immunotherapy has reshaped the traditional treatment paradigm and become the new standard of care for patients with resectable stage II and III NSCLC.^18,19^ However, the dissemination and applicability of this novel therapeutic strategy have been accompanied by several challenges, namely the generalizability of trial outcomes to the real-world populations, and the influence of this regimen on surgical complexity and perioperative patient management.^20^ Our study is the first nationwide study from Europe with full participation of all accredited thoracic oncology centers reporting real-world surgical practice patterns and outcomes during the first year of implementation of this novel treatment in the Czech Republic. This study adds to the current literature of randomized clinical trials and previously published real-world data.

Compared with CheckMate816^8^, our real-world cohort included a higher proportion of patients aged ≥ 65 years (73.4% vs. 48.0%), more female patients, and more patients with ECOG performance status 1, whereas histology and smoking history were similar. Direct comparison of stage distribution should be interpreted with caution, as our study used the AJCC 8th edition staging system, and CheckMate816 used the AJCC 7th edition. Completion of all three neoadjuvant cycles was comparable to that in CheckMate816, and the rate of failure to proceed to surgery was not increased (12.5% vs. 15.6%). Importantly, the inclusion of patients who did not proceed to surgery allowed us to capture reasons for treatment discontinuation and subsequent therapeutic management in a real-world setting. Delayed surgery was more frequent in our cohort (39.3% vs. 20.8%). In both studies, most delays were < 2 weeks and were predominantly administrative. Median operative time was similar (167.5 vs. 185 min). Open thoracotomy remained the most common approach, with minimally invasive surgery attempted in comparable proportions. However, conversion rates were lower in our cohort (11.5% vs. 27.9%). Lobectomy was the predominant procedure in both studies, whereas pneumonectomy was less frequent in our cohort (8.9% vs. 16.8%). The median length of stay was slightly shorter than in CheckMate816 (7 vs. 10 days). The R0 resection rate was higher in our nationwide cohort (98.2% vs. 83.2%), with a comparable lymph node yield. When comparing our ITT population, in which all patients had PD-L1 expression > 1%, with the PD-L1–positive subgroup from CheckMate816, both pCR (40.6% vs. 32.6%) and MPR (48.4% vs. 44.9%) rates were comparable.

Until recently, the real-world evidence on surgical outcomes in patients treated with neoadjuvant chemo-immunotherapy according to the CheckMate816 protocol has been limited. The largest European surgical cohort was reported by Brunelli et al.^19^ in 2025 and included 340 patients (215 with PD-L1 ≥ 1%). Demographic characteristics, including a higher proportion of older patients, were similar to those in our cohort. Failure to proceed to surgery was lower (6.8%) than the 16–23% range reported in phase III trials.^8–11^ Delayed surgery was more frequent in real-world cohorts (47.0% and 39.3% in Brunelli et al.^19^ and our study, respectively) than in CheckMate816 (20.8%). The association between surgical delay and pCR has not yet been observed,^8,19^ and the true impact of surgical delay on operative complexity has likewise not been well defined.^21^ Across real-world series, lobectomy remains the predominant type of resection, with pneumonectomies being uncommon (2–5%), in contrast to the pneumonectomy rate of 9–17% reported in clinical trials.^17–19,22–24^ Minimally invasive surgery after neoadjuvant chemo-immunotherapy has been reported as safe and feasible in high-volume centers, with reported rates of 65.6–95% (predominantly VATS) and conversion rates of 5–18.7%.^17–19,22,23^ Higher conversion rates were observed for VATS than for RATS (28.2% vs. 7.5%), potentially reflecting differences in surgical platform flexibility and visualization. Interestingly, a greater number of lymph nodes have been harvested with RATS, with potential implications for pathological assessment, staging accuracy, and subsequent treatment decisions.^25^

Following the introduction of neoadjuvant chemo-immunotherapy for resectable NSCLC, one of the central questions has been its impact on surgical complexity and the ability to achieve complete resection. R0 resection rates range from 83.2 to 95.8% in clinical trials and consistently exceed 90% in real-world series, including our cohort.^8–11,17–19,22–27^ Surgery may be technically more demanding because of perihilar fibrosis and inflammation, particularly in patients with cN1 disease or marked nodal response. Importantly, patients selected for neoadjuvant chemo-immunotherapy may already present increased technical challenges at baseline.^24,28^ However, reliable clinical, laboratory, or radiographic predictive biomarkers of intraoperative difficulty remain limited, and despite several proposed frameworks, standardized and reproducible scoring systems for quantification of intraoperative challenges are lacking.^13,29,30^ Reported 90-day postoperative mortality ranges from 1.3 to 4% in trials and from 2.5 to 5.8% in real-world practice, potentially reflecting the greater clinical complexity and comorbidity.^8–11,17–19,23,26,27^ In our cohort, 90-day mortality was 5.4%. Although this was higher than that reported in the Czech national registry, this finding should be interpreted cautiously because of the small sample size and the absence of deaths directly attributable to the surgical procedure.

Pathological response endpoints are recognized surrogate markers for long-term survival in resectable NSCLC.^29^ In phase III trials, pCR and MPR rates have ranged from 17.2 to 40.7% and from 32.2 to 56.2%, respectively.^8–11,26,27^ In real-world ITT populations, pCR and MPR rates have generally ranged from 17 to 29.2% and from 30 to 52.7%, respectively, and from 22.2 to 30.5% and 42.3 to 55.3% among patients with PD-L1 expression ≥ 1%. However, several real-world series reported a substantial proportion of patients with missing or unknown PD-L1 status.^17–19,22,23^ Our cohort consisted exclusively of patients with PD-L1 expression ≥ 1%, in whom pCR and MPR rates in the ITT population were 40.6% and 48.4%, respectively. Several factors may contribute to these findings. Although CheckMate816 demonstrated a benefit of neoadjuvant chemo-immunotherapy regardless of PD-L1 status, the magnitude of benefit and depth of pathological response was greater in patients with PD-L1 expression ≥ 1%.^8,13^ All patients in our study underwent PD-L1 testing, as PD-L1 positivity was an inclusion criterion, resulting in complete data for this variable, whereas a substantial proportion of previously published real-world cohorts reported missing PD-L1 information.^17–19,22,23^ Finally, the absence of centralized pathological review in our study reflects real-world clinical practice, in which pathological response assessment may vary across institutions and could have influenced reported pCR and MPR rates, although it is known that there is a very high agreement on pCR among pathologists. This heterogeneity also resulted in missing residual viable tumor data and, consequently, unavailable MPR assessment in five patients (8.9%) from one participating center. These observations further underscore the need for standardized pathological evaluation to improve the reproducibility of outcomes following neoadjuvant chemo-immunotherapy. Beyond the early adoption phase, future efforts will focus on refinements of perioperative treatment strategies. Real-world evaluation of different immunotherapy regimens with varying treatment intensity and/or number of cycles will be critical to balance oncological benefit, surgical feasibility, and cost effectiveness.^31^

Several limitations should be considered when interpreting the results of this study. First, the observational design precludes causal conclusions and provides complementary real-world evidence to findings from randomized phase III trials. The study population was drawn from multiple centers, with varied patient enrolment across participating institutions, reflecting differences in case volume and institutional experience inherent to real-world practice. Despite the nationwide nature of the cohort, the number of operated patients during the early adoption phase limited the statistical power for detailed subgroup analyses. Accordingly, subgroup comparisons by clinical stage, PD-L1 expression, histological subtype, and nodal status should be interpreted with caution and considered exploratory and, at best, hypothesis-generating. Finally, follow-up duration was limited, reflecting the early phase of real-world implementation of neoadjuvant chemo-immunotherapy in the Czech Republic, and the low number of recurrence events precludes meaningful assessment of recurrence patterns, event-free survival, or long-term oncological outcomes.

## Conclusion

This nationwide, multi-institutional real-world study demonstrated the safe and successful implementation of a novel therapeutic strategy into clinical practice. During the early adoption period, nivolumab-based neoadjuvant chemo-immunotherapy for resectable NSCLC was associated with perioperative outcomes comparable to those from seminal clinical trials and high R0 resection rates, despite perceived increased surgical complexity. Our findings are consistent with recently reported real-world surgical outcomes following neoadjuvant chemo-immunotherapy with nivolumab and support its continued integration into routine clinical practice worldwide.

## Supplementary Information

Below is the link to the electronic supplementary material.

Supplementary file1 (DOCX 21 kb)
