# Supplementary material for: Nationwide Implementation of Neoadjuvant Nivolumab-Based Chemo-Immunotherapy for Resectable NSCLC: Surgical Outcomes from a Prospective Multicenter Study
Source: Ann Surg Oncol. 2026 Jul 30;33(12):10312–25. doi: 10.1245/s10434-026-20310-2 (PMC13675273; doi:10.1245/s10434-026-20310-2)
Supplement: Supplementary file 1 [file 10434_2026_20310_MOESM1_ESM.docx]

**Supplementary data:**

**Nationwide Implementation of Neoadjuvant Nivolumab-Based Chemo-Immunotherapy for Resectable NSCLC: Surgical Outcomes from a Prospective Multicenter Study**

**METHODS**

*Treatment pathway and clinical practice*

Patient distribution among the participating centers was as follows: Prague - University Hospital Motol 21, Olomouc 12, Prague - Thomayer University Hospital 10, Brno 6, Ústí nad Labem 5, Hradec Králové 4, Ostrava 3, and Plzeň 3. Each multidisciplinary tumour board (MDT) consisted of pulmonologists, medical oncologists, radiation oncologists, radiologists, and thoracic surgeons. Invasive mediastinal staging was performed according to local institutional practice at each accredited center, as no standardized nationwide approach currently exists in the Czech Republic. Restaging with PET/CT or CT was routinely scheduled after administration of the final dose of chemo-immunotherapy. Imaging results were subsequently reviewed by a re-evaluation MDT according to RECIST version 1.1 or PERCIST criteria.^1,2^ All procedures were performed by a surgical team that included a board-certified thoracic surgeon. Selection of the surgical approach was solely at the discretion of the thoracic surgeon, based on preoperative imaging and intraoperative findings. Complete (R0) resection was assessed according to the International Association for the Study of Lung Cancer (IASLC) definition.^3^ Intraoperative technical complexity characteristics, as described by Feldman et al. were recorded.^4^ Postoperative complications were recorded using the Clavien–Dindo classification during the first 30 days after surgery and within 30 days after hospital discharge.^5^ At the principal investigator’s site, pathological assessment and evaluation of pathological response to treatment were performed according to the IASLC multidisciplinary recommendations.^6^ In other participating centers, pathological evaluation was performed according to institutional protocols and routine clinical practice. No centralized pathological review, study-specific pathology training, or inter-institutional quality control measures were implemented, as the study was designed to capture real-world clinical practice in the early adoption phase rather than mandate standardized pathological assessment across participating centers.

*Variables collected and used for the analysis*

Patient demographics, including age at the time of diagnosis, sex, performance status, smoking history, body mass index, and history of previous thoracic surgery; histological subtype; initial clinical stage; and PD-L1 tumour proportion score (TPS). Baseline pulmonary function parameters, including FEV1 and DLCO, were not included in the predefined dataset and therefore were not systematically collected across participating centers. Regarding neoadjuvant treatment and restaging, following variables were collected: type of chemotherapy, number of completed treatment cycles, treatment-related adverse events (TRAEs) leading to surgery delay or cancellation, type of restaging imaging modality, and radiological response status. Surgical variables included the interval from administration of the last dose of neoadjuvant treatment to surgery, attrition to surgery, reasons for surgical cancellation, operative time, surgical approach (including conversion from minimally invasive to open surgery), anatomical extent of resection, intraoperative technical complexity characteristics, and estimated blood loss. Postoperative surgery-related adverse events, as defined by Fernández et al., were recorded within 30 days after surgery or later if the patient remained hospitalized.^7^

**SUPPLEMENTARY TABLE S1 Intraoperative technical complexity**

| **Characteristics** | **Statistics** | **Patients with surgery  (n=56)** |
| --- | --- | --- |
| Intrapericardial dissection | n (%) | 4 (7.1) |
|  | | |
| Necessity of chest wall resection | n (%) | 3 (5.4) |
|  | | |
| *↳* *Extent of chest wall resection* | *extrapleural resection, n (%)* | *1 (33.3)* |
|  | *3 ribs, n (%)* | *1 (33.3)* |
|  | *4 ribs, n (%)* | *1 (33.3)* |
|  | | |
| Necessity of reconstruction (sleeve or bronchoplasty) because of central tumor involvement | n (%) | 6 (10.7) |
|  | | |
| *↳* *Type of reconstruction* | *Bronchial, n (%)* | *3 (50.0)* |
|  | *Vascular, n (%)* | *1 (16.7)* |
|  | *Both, n (%)* | *2 (33.3)* |
|  | | |
| Intraoperative blood loss [mL] | Median (IQR) | 175 (60-325) |
|  | | |
| RBC [units] | 0, n (%) | 52 (92.8) |
|  | 1, n (%) | 1 (1.8) |
|  | 2, n (%) | 3 (5.4) |
|  | | |
| Whole Blood [units] | 2, n (%) | 1 (1.8) |
|  | | |
| Significant fibrosis leading to technical difficulties | n (%) | 28 (50.0) |
|  | | |
| Significant lymphadenopathy (matted / sticky / hard) leading to technical difficulties | n (%) | 25 (44.6) |
|  | | |
| Lymph nodes unable to be removed from the pulmonary artery | n (%) | 7 (12.5) |

| Lymph node adherence to the pulmonary artery resulting in tear | n (%) | 4 (7.1) |
| --- | --- | --- |
|  | | |
| Proximal and / or intra-pericardial pulmonary artery control required because of nodal adherence | n (%) | 9 (16.1) |
|  | | |
| Change in extent of resection because of lymph nodal adherence to structures | n (%) | 4 (7.1) |
|  | | |
| Pulmonary arterioplasty or sleeve resection only related to nodal adherence | n (%) | 4 (7.1) |
|  | | |
| Change in approach to vasculature (switch from artery first to vein first and vice versa) because of encountering difficulty related to nodal adherence | n (%) | 7 (12.5) |

Data are presented as counts and percentages or as median with interquartile range, as appropriate. For italicized subcategories, percentages are calculated using the total number of patients within the respective parent subgroup as the denominator. Details of chest wall resection are provided exclusively for affected patients and include the number of ribs resected, including extrapleural resection without rib removal where applicable. Intraoperative reconstructive procedures comprise bronchial, vascular, or combined sleeve or plasty techniques performed for central tumor involvement. Blood product use is expressed as the number of units administered intraoperatively. Fibrosis and lymphadenopathy refer to surgeon-assessed intraoperative findings associated with increased technical difficulty. Abbreviations: RBC, red blood cells; IQR, interquartile range.

**Supplementary references:**

S1. Eisenhauer EA, Therasse P, Bogaerts J, Schwartz LH, Sargent D, Ford R, et al. New response evaluation criteria in solid tumours: revised RECIST guideline (version 1.1). Eur J Cancer. 2009;45(2):228-47. <https://doi.org/10.1016/j.ejca.2008.10.026>

S2. O JH, Lodge MA, Wahl RL. Practical PERCIST: A Simplified Guide to PET Response Criteria in Solid Tumors 1.0. Radiology. 2016;280(2):576-84. <https://doi.org/10.1148/radiol.2016142043>

S3. Rami-Porta R, Wittekind C, Goldstraw P, International Association for the Study of Lung Cancer Staging C. Complete resection in lung cancer surgery: proposed definition. Lung Cancer. 2005;49(1):25-33. <https://doi.org/10.1016/j.lungcan.2005.01.001>

S4. Feldman HA, Zhou N, Deboever N, Hofstetter W, Mehran R, Rajaram R, et al. Intraoperative challenges after induction therapy for non-small cell lung cancer: Effect of nodal disease on technical complexity. JTCVS Open. 2022;12:372-84. <https://doi.org/10.1016/j.xjon.2022.09.012>

S5. Clavien PA, Barkun J, de Oliveira ML, Vauthey JN, Dindo D, Schulick RD, et al. The Clavien-Dindo classification of surgical complications: five-year experience. Ann Surg. 2009;250(2):187-96. <https://doi.org/10.1097/sla.0b013e3181b13ca2>

S6. Travis WD, Dacic S, Wistuba I, Sholl L, Adusumilli P, Bubendorf L, et al. IASLC Multidisciplinary Recommendations for Pathologic Assessment of Lung Cancer Resection Specimens After Neoadjuvant Therapy. J Thorac Oncol. 2020;15(5):709-40. <https://doi.org/10.1016/j.jtho.2020.01.005>

S7. Fernandez FG, Falcoz PE, Kozower BD, Salati M, Wright CD, Brunelli A. The Society of Thoracic Surgeons and the European Society of Thoracic Surgeons general thoracic surgery databases: joint standardization of variable definitions and terminology. Ann Thorac Surg. 2015;99(1):368-76. <https://doi.org/10.1016/j.athoracsur.2014.05.104>
